# The Efficiency of Homologous Recombination and Non-Homologous End Joining Systems in Repairing Double-Strand Breaks during Cell Cycle Progression

**DOI:** 10.1371/journal.pone.0069061

**Published:** 2013-07-11

**Authors:** Leonardo Bee, Sonia Fabris, Roberto Cherubini, Maddalena Mognato, Lucia Celotti

**Affiliations:** 1 Dipartimento di Biologia, Università di Padova, Padova, Italy; 2 Laboratori Nazionali di Legnaro, Istituto Nazionale di Fisica Nucleare, Padova, Italy; St. Georges University of London, United Kingdom

## Abstract

This study investigated the efficiency of Non-Homologous End Joining (NHEJ) and Homologous Recombination (HR) repair systems in rejoining DNA double-strand breaks (DSB) induced in CCD-34Lu cells by different γ-ray doses. The kinetics of DNA repair was assessed by analyzing the fluorescence decrease of γ-H2AX foci measured by SOID (Sum Of Integrated Density) parameter and counting foci number in the time-interval 0.5–24 hours after irradiation. Comparison of the two methods showed that the SOID parameter was useful in determining the amount and the persistence of DNA damage signal after exposure to high or low doses of ionizing radiation. The efficiency of DSB rejoining during the cell cycle was assessed by distinguishing G1, S, and G2 phase cells on the basis of nuclear fluorescence of the CENP-F protein. Six hours after irradiation, γ-H2AX foci resolution was higher in G2 compared to G1 cells in which both NHEJ and HR can cooperate. The rejoining of γ-H2AX foci in G2 phase cells was, moreover, decreased by RI-1, the chemical inhibitor of HR, demonstrating that homologous recombination is at work early after irradiation. The relevance of HR in DSB repair was assessed in DNA-PK-deficient M059J cells and in CCD-34Lu treated with the DNA-PKcs inhibitor, NU7026. In both conditions, the kinetics of γ-H2AX demonstrated that DSBs repair was markedly affected when NHEJ was absent or impaired, even in G2 phase cells in which HR should be at work. The recruitment of RAD51 at DSB sites was, moreover, delayed in M059J and in NU7026 treated-CCD-34Lu, with respect to DNA-PKcs proficient cells and continued for 24 hours despite the decrease in DNA repair. The impairment of NHEJ affected the efficiency of the HR system and significantly decreased cell survival after ionizing radiation, confirming that DSB rejoining is strictly dependent on the integrity of the NHEJ repair system.

## Introduction

It is known that exposure to ionizing radiation (IR) causes many types of DNA damage, and, among these, double-strand breaks (DSBs) are considered the most dangerous threat to genomic integrity [1], [2]. Radio-induced DSBs can have a different complexity with respect to the ionization density of radiation. It has been demonstrated that high-LET radiation induces clusters of DNA lesions along the particle track while low-LET radiation causes sparse ionizations. When administered at high doses, low-LET radiation can also, nevertheless, lead to complex DNA damage [3] consisting of DSBs associated with base damages as well as non-DSB damage clusters comprised of base lesions, apyrimidinic or apurinic sites and single-strand breaks that can produce additional DSBs due to damage processing [4].

The efficiency of DNA repair after exposure to IR depends on the complexity of the radio-induced damage [5]. The presence of DSBs, whatever their origin may be, elicits a complex DNA-Damage Response (DDR) consisting of a cascade of events, involving damage sensing, signal transduction to the effectors of DNA repair, cell cycle arrest, and induction of apoptosis [6]. After exposure to IR, the extensive phosphorylation of histone H2AX at Ser139 results in the formation of discrete γ-H2AX foci which can be easily identified by immunostaining, a valuable tool highlighting the presence of DSBs [7], [8]. Since phosphorylation of H2AX at Ser 139 is abundant, fast, and correlates well with each DSB, it is the most sensitive marker that can be used to examine DNA damage and subsequent lesion repair [9]. Apart from γ-H2AX, numerous additional proteins that participate in DDR form Ionizing Radiation Induced Foci (IRIF) through their recruitment and accumulation at DNA damaged sites and often closely overlap with the relatively large γ-H2AX foci. One of these, the tumor suppressor p53-binding protein 1 (53BP1) rapidly localizes at DSB sites and activates p53 along with specific kinases. The number of 53BP1 foci has a linear relationship with the irradiation dose, and the time course of 53BP1 foci formation and disappearance is similar to that of γ-H2AX foci [10]–[14]. Another, smaller type of foci restricted to stretches of single-stranded (ss) DNA produced from DSB end resection is formed by the components of the homologous recombination (HR) repair pathway, including Rad51 and RPA proteins. RPA binds to ssDNA during the initial phase of homologous recombination. Just as in DNA replication, this keeps ssDNA from binding to itself, in such a way that the resulting nucleoprotein filament can then be bound by Rad51 and its cofactors [15]. Broadly similar to the γ-H2AX foci detection, these additional foci provide convenient surrogate markers useful for monitoring the presence of DNA DSBs or the recruitment of HR repair proteins.

Eukaryotic cells rely on two highly regulated DSB repair pathways: the non-homologous end joining (NHEJ) and homologous recombination (HR). The former, which rejoins the DNA ends without requiring sequence homologies, is carried out by the DNA-dependent protein kinase (DNA-PK) holoenzyme, consisting of the heterodimer KU70/KU80 and the DNA-PK catalytic subunit (DNA-PKcs) and by the DNA LIG4-XLF (Cernunnos)-XRCC4 complex. HR’s central activity is coordinated by RAD51 protein, which catalyzes the strand capture and invasion of broken ends of DSBs into intact homologous DNA sequences, which are the sister chromatid or the homologous chromosome, to ensure the fidelity of the repair process [16]–[19]. Although both NHEJ and HR contribute to DSB rejoining, their involvement varies during the different cell cycle phases as NHEJ is active throughout all cell cycle while HR is active during the S and G2 phases when sister chromatids are available. Some authors observed that the cell cycle control of DSB pathway choice can be bypassed in IR-exposed cells, thus promoting a preferential repair by HR [20]–[22].

The involvement and efficiency of NHEJ and HR repair systems during cell cycle phases in normal human CCD-34Lu fibroblasts exposed to different γ-ray doses were analyzed here. The study aimed, moreover, to determine if the impairment of DNA-PKcs protein by NU7026, a chemical inhibitor, or due to a frameshift mutation in M059J cells, alters RAD51 protein activity during the repair of γ-ray-induced DSBs. In order to analyze DSB repair at different stages of the cell cycle, G1and G2 phases were distinguished on the basis of the nuclear fluorescence intensity of CENP-F protein, whose expression and localization are cell cycle-dependent. CENP-F is a protein of the nuclear matrix that gradually accumulates during the cell cycle until it reaches peak levels in G2 and M phase cells and is rapidly degraded after mitosis is complete [23]. It is thus detectable by *in situ* immunofluorescence throughout the late S, G2, and M phases of the cell cycle, but it is absent in the G1 one [24].

We used different methods based on quantifying foci fluorescence as an indicator of DNA damage and repair to study the kinetics of DNA DSB rejoining during the cell cycle. From our experiments, the Sum Of Integrated Density (SOID) parameter [25] results a valuable tool which takes into account the number and the size of IR-induced foci, allowing to accurately quantify DNA damage signal after exposure to high or low doses of ionizing radiation [26].

Our data indicated that the NHEJ and HR repair systems cooperate in G2 phase cells in DSB rejoining not only long after irradiation, but also during the first hours of post-irradiation incubation. We also noted that besides decreasing the general efficiency of DNA repair, the impairment of NHEJ in CCD-34Lu treated with the DNA-PKcs inhibitor, NU7026, as well as in DNA-PKcs deficient M059J cells likewise affected RAD51 recruitment to DSB sites.

## Materials and Methods

### Cell Lines

Normal human neonatal lung fibroblasts CCD-34Lu (ATCC N. CRL-1491™) were grown in high glucose (4.5 g/l) Dulbecco’s Modified Eagle Medium (DMEM) containing GlutaMAX (Gibco, Life Technologies), supplemented with 10% heat-inactivated fetal calf serum (FCS, Biochrom KG, Seromed), HEPES 20 mM (Sigma-Aldrich), 1% MEM non-essential amino acids (Gibco, Life Technologies). At the time the experiments were carried out the cells were at 27 to 40 population doublings and actively proliferating, as confirmed by flow cytometry analysis. Human malignant M059J glioblastoma cells were purchased from ATCC (CRL-2366™), while M059K cells were kindly provided by Professor S.C. West (Cancer Research UK London Research Institute, Clare Hall Laboratories, South Mimms, UK). Both cell lines were grown in a 1∶1 mixture of DMEM and Ham’s F-12 medium (DMEM/F-12, Gibco, Life Technologies), HEPES 20mM, 1% of MEM non-essential amino acids and 10% of heat-inactivated FCS.

### Cell Irradiation

Gamma irradiation was performed at the Department of Oncological and Surgical Sciences of the University Padova Medical Center with a ^137^Cs source (dose rate of 2.8 Gy/min). Cells (0.4×10^6^) were seeded 48h before the experiment and irradiated on Petri dishes (60×15 mm), with or without coverslips, kept on ice before and after irradiation, and cultured at 37°C in fresh medium for different repair times. Except for irradiation, the control cells were subjected to the same experimental conditions.

### Immunofluorescence Staining

The cells were fixed at 0.5, 2, 6 and 24h after irradiation for Fluorescence Intensity (FI) analyses. Non-irradiated and irradiated cells were rinsed once with cold Phosphate Buffered Saline (PBS) and fixed with a 4% solution of formaldehyde (Sigma-Aldrich) at 37°C for 15 min and washed three times with PBS. The cells were permeabilized with 0.5% Triton X-100 in PBS at 37°C for 10 min and non-specific binding sites were masked with goat serum (10% in PBS) at room temperature for 1h. The cells were incubated for 2h at room temperature with anti-53BP1 (Bethyl Laboratories, 1∶100), anti- γ-H2AX (Ser139) (Abcam or Millipore Chemicon Upstate Clone JBW301, 1∶100), anti-RAD51 (Santa Cruz Biotechnology, H-92: sc-8349, lot. G0811, 1∶100), anti–CENP-F (BD Bioscience, 610768, Clone 11, 1∶100 or Abcam, ab5, lot. GR73067-3, 1∶300) primary antibodies followed by three washings in PBS and once in PBS +0.1% Triton X-100. The cells were subsequently incubated at room temperature for 1h with Alexa Fluor 488 goat anti-mouse secondary antibodies and Alexa Fluor 594 donkey anti-rabbit antibodies (Life Technologies, 1∶250 and 1∶350, respectively) and washed, as described above. Immunofluorescent staining for RPA and R2 were performed using the MAX-Stain™ reagents (Active Motif) according to the manufacturer’s instructions. Briefly, fixed cells were incubated for 1h at 37°C with MAX-Block™ Blocking medium, washed 10 min with PBS, and incubated for 1h at 37°C with primary anti-R2 (Santa Cruz, N-18 sc:10844, lot. K061, 1∶200) and anti-RPA32/RPA2 antibodies (Abcam, 9H8 lot. GR92538-1, ab2175, 1∶200) diluted in MAX-Bind™ staining medium. The cells were then washed three times in PBS +0.05% Tween-20, incubated for 1h at 37°C with secondary antibodies diluted in MAX-Bind™ staining medium, and washed, as described above. Cover slips were then mounted on glass slides with Vectashield mounting medium (Vector Laboratories) containing DAPI 0.2 µg/ml.

Images of 53BP1, γ-H2AX and RAD51 and *RPA* foci were taken using a Leica TCS SP5 confocal microscope (Leica Microsystems) with 40× or 63× oil immersion objectives. All images were acquired under the same laser intensity, PMC voltage, pinhole aperture, and 8-bit intensity value conditions. Z-plane stack scanning (500 nm thickness) was performed using sequential scanning to prevent crosstalk due to overlap of the emission spectra from the various fluorophores. Manual counts of γ-H2AX and 53BP1 foci were performed using the maximum intensity projection (MIP) images. The red and green images were superimposed by ImageJ software (NIH) to obtain merged images. The number of γ-H2AX and 53BP1 foci was determined for each time point on an average of 100 nuclei in three independent experiments and listed in the figures after the number of foci in the non-irradiated cells was subtracted.

### Nocodazole Treatment

We added the spindle poison nocodazole (Sigma-Aldrich) to CCD-34Lu to arrest cell cycle progression during the M phase 1h before irradiation at 5 and 10 Gy at the final concentration of 50 ng/mL. After irradiation the cells were maintained for 2h in the drug’s presence and then analyzed by immunofluorescence for the presence of CENP-F nuclear protein and RAD51 foci.

### Quantification of Nuclear Fluorescence

The images acquired with the confocal microscope were processed and analyzed with ImageJ software, using a specifically designed Macro to enable automated analysis of a larger number of nuclei (average of 200 nuclei) for each time point. All the images were processed to remove the background. The nuclear area for each image was determined by 4′, 6-Diamidino-2-phyenylindole (DAPI) fluorescent signal and saved as a list of coordinates for subsequent analyses. Nuclear fluorescence was calculated as the mean intensity of all the pixels included in the nuclear area. In accordance with Mistrik et al. [25] and Ishikawa et al. [26], the SOID parameter was calculated for each nucleus as the product of the sum of the area of the foci and the mean fluorescence intensity. An intensity threshold was set to calculate the SOID so that only foci were included in the analysis.

The nuclear fluorescence intensity (FI) of CENP-F protein was used to discriminate the γ-H2AX and RAD51 SOID signal in the G2 and G1 cells. We also assigned the specific range of CENP-F FI values to G1, S, and G2 cells correlating CENP-F nuclear FI with the DNA replication phase using the EdU (5-ethynyl-2-deoxyuridine, Life Technologies) staining method described by Salic et al. and Buck et al. [27], [28], with minor modifications. Non-irradiated cells were seeded on Petri dishes with glass coverslips 48h before labeling for EdU assay. The cells were then incubated with EdU (30 µM) for 1h, rinsed three times with PBS and fixed with 4% of formaldehyde for 15 min at 37°C. The cells were washed again before the “Click” stain reaction was performed and permeabilized with Triton X-100 0.5% in TBS for 5 min at RT. The “Click” reaction was performed incubating the cells for 30 min with a freshly prepared mix of 50 mM Tris-HCl pH 7.3, 2 mM CuSO_4_, 5 µl/ml fluorescent 647-azide, 10 mM ascorbic acid and used immediately after ascorbate was added. EdU-stained coverslips were immunostained with CENP-F antibody, as described. Double stained slides were acquired using a Leica TCS SP5 confocal microscope and nuclear fluorescence was quantified. The range of CENP-F FI associated with the 95% of EdU positive cells identifying S-phase cells was calculated while the CENP-F FI values associated with EdU negative nuclei were assigned to the G1 cells. Finally, the values of CENP-F FI associated with EdU negative nuclei but higher than the maximum value of S-phase cells were assigned to the G2 cells. Throughout the different cell cycle phases during analyses of DNA repair we excluded nuclei with CENP-F intensity values within the confidence interval of S-phase, estimated as the mean CENP-F values ± S.D.

Due to the incompatibility between CENP-F and RPA antibodies, we used the presence of cytosolic RPA fluorescence of the ribonucleotide reductase R2 subunit as a marker of G1 and G2 phases [29]. R2 positive cells (S-G2) were discriminated by the presence of cytosolic fluorescence.

### FACS Analyses

The cell cycle distribution of irradiated and non-irradiated control cells was assessed by flow cytometry analysis of DNA content following staining with 50 µg/µl of propidium iodide (PI, Sigma-Aldrich), as previously described [30].

To analyze CENP-F content throughout the cell cycle, the cells were fixed in 70% cold ethanol, rinsed twice in PBS, centrifuged at 200 g for 10 min at 4°C, and permeabilized in PBS with 0.1% TritonX-100 and 4% goat serum for 10 min on ice. After centrifugation, the cells were incubated over night with primary antibody diluted in permeabilization solution (mouse anti-CENP-F, 1∶100). Then the cells were rinsed three times in PBS with 2% of goat serum and incubated at room temperature for 1h with agitation with secondary antibody (Alexa Fluor 488 goat anti-mouse) diluted in permeabilization solution. After three washings in PBS with 2% goat serum, the cells were stained with at 37°C for 1h. FACS analysis of total γ-H2AX content was carried out in a similar way, using a rabbit anti-γ-H2AX (1∶500) as the primary antibody and Alexa Fluor 488 goat anti-mouse as the secondary one.

Data concerning FI were collected from 10×10^3^–25×10^3^ cells/sample using a BD FACSCanto™ II flow cytometer (Becton Dickinson, BD Biosciences) and analyzed using the ModFit LT software (Verity Software House).

### NU7026 and RI-I Treatments

To specifically inhibit NHEJ or HR, 24h before irradiation CCD-34Lu cells were incubated with 10 µM NU7026 (DNA-PKcs inhibitor, Sigma-Aldrich) or 10 µM of RI-1 (RAD-51 inhibitor, CALBIOCHEM), both diluted in DMSO. After irradiation, the medium was replaced with a fresh one containing the inhibitor, and the cells were incubated for the fixed repair times. Non-irradiated cells were treated with DMSO only, NU7026 only, or RI-1 only to exclude any potential toxicity from contributing to the effects of radiation; no differences were detected in the various treatment conditions.

### Cell Viability

Cell viability was determined by a clonogenic assay in non-irradiated and irradiated CCD-34Lu, incubated with or without NU7026, the DNA-PKcs inhibitor, in M059K and M059J cells. After irradiation, 200 viable CCD34-Lu cells were seeded together with feeder layer cells (IMR90, 15×10^5^ cells/plate), previously irradiated with 40 Gy of γ-rays in complete medium supplemented with 15% serum in 10 cm diameter Petri dishes. When CCD-34Lu cells were treated with NU7026 they were maintained for 24h with the inhibitor and then the medium was replaced with a fresh one without the inhibitor. During clonogenic assay, 500 viable M059K and M059J cells were seeded in complete medium, without a feeder layer. Culture plates were scored for colony formation 14 days later by staining cells with crystal violet 0.4%. Only colonies containing at least 50 cells were considered positive. Cell survival was calculated as the percentage of cloning efficiency of treated cells over that of control cells.

### Statistical Analysis

Data from at least three separate experiments are presented as means ± standard deviation (S.D.). All comparisons, with the exception of the cell survival experiments, were calculated using Student’s *t*-test, in which case the *P* values are based on a two-way ANOVA analysis. Differences with a <0.05 *P*-value are considered significant.

## Results

### Kinetics of the Formation and Repair of DNA Double-strand Breaks

**Figure 1:**
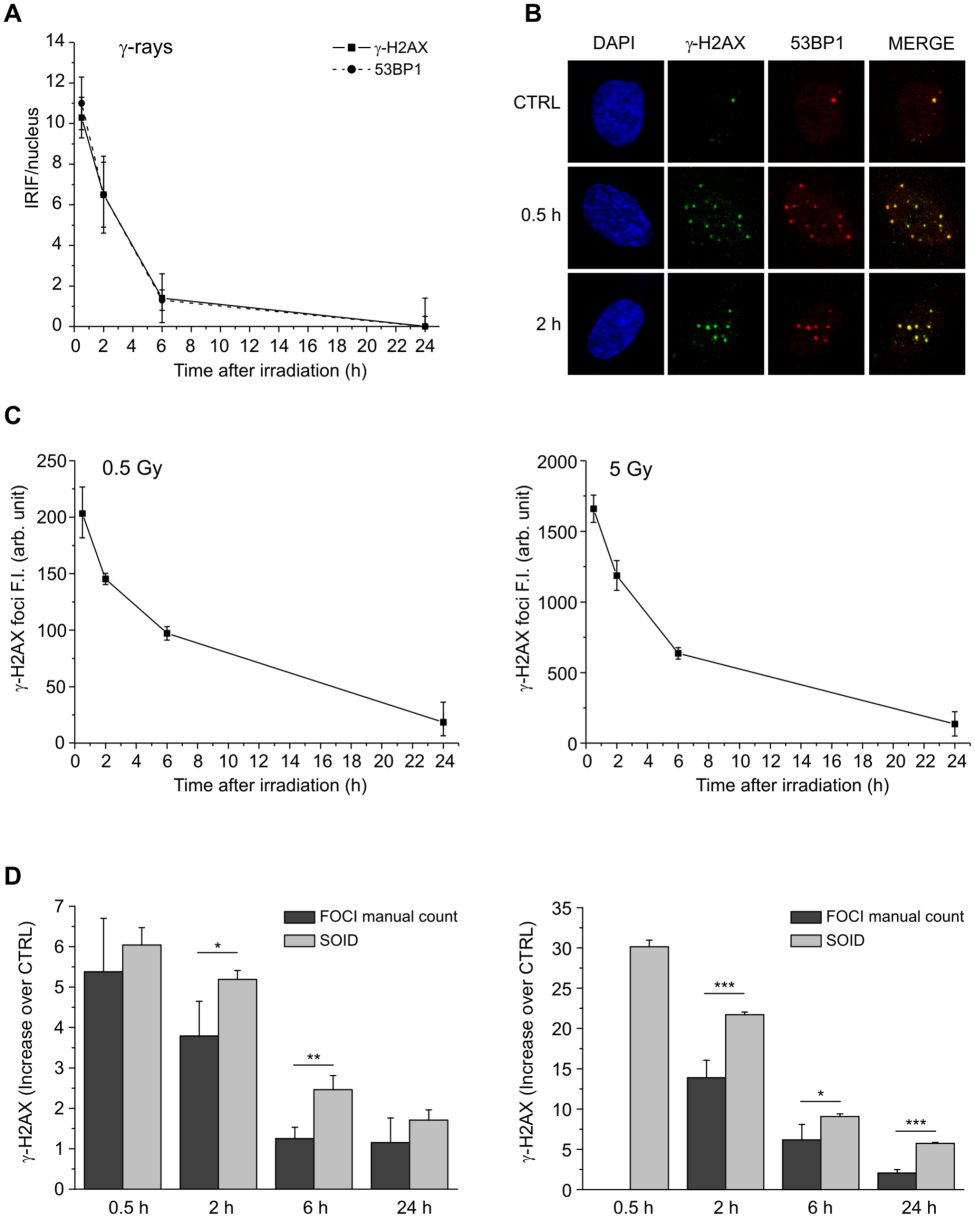
Kinetics of DSB rejoining in cells irradiated with γ-rays plotted against 24h of the repair time. (A) DBS rejoining was measured by manual counting of γ-H2AX and 53BP1 foci induced by irradiation with 0.5 Gy. (B) Representative pictures of γ-H2AX (green) and 53BP1 foci (red) in nuclei (blue) at 0.5 and 2 h from γ-irradiation, showing the almost complete co-localization of γ-H2AX and 53BP1 foci. (C) Fluorescence intensity (FI) of γ-H2AX foci has been determined by SOID parameter in cells irradiated with 0.5 Gy and 5 Gy of γ-rays. (D) Comparison of FI of γ-H2AX foci, induced by irradiation with 0.5 Gy and 5 Gy, analyzed by manual counting and SOID parameter, expressed as increase over control. Error bars represent standard deviation of the mean calculated from 3–4 experiments (*P<0.05, **P<0.01, ***P<0.001, SOID vs. foci number, Student’s *t*-test).

The formation and rejoining of DNA DSBs were analyzed by determining the number of ionizing radiation induced foci (IRIF) of γ-H2AX and 53BP1 proteins in CCD-34Lu cells irradiated with 0.5 Gy of γ-rays. Our data indicated that the kinetics of DSB rejoining is characterized by a complete resolution of IRIF within 24h of irradiation and the almost complete co-localization of γ-H2AX and 53BP1 foci (Figure 1A, B). Although the number of foci is correlated with the number of DNA DBSs [9], this parameter alone cannot precisely quantify the amount of DNA damage signal, which is linked to the size and persistence of foci during DNA-repair kinetics. The SOID parameter, which accounts for the number, the size, and the fluorescence density of ionizing radiation-induced foci, was thus utilized to accurately quantify the DNA damage. The results obtained, indicated in the text and in the figures as foci fluorescence intensity (FI), showed that the resolution of foci in cells irradiated with 0.5 and 5 Gy occurred with similar kinetics for both doses, even if with different values, according to the dose-related intensity of DNA damage signal (Figure 1C). By comparing the results of γ-H2AX kinetics obtained on the same samples of irradiated cells by manual foci counting and SOID parameter, we observed that in 0.5 Gy irradiated cells the kinetics of DNA damage signal (SOID parameter) and the DSB resolution (foci number) were rather similar, as both methods showed a complete DBS resolution in the 0.5–24h time-interval (Figure 1D). Following irradiation with 5 Gy, it was impossible to count the foci manually as shortly after irradiation the foci number was too high for reliable eye resolution. As a result we could not compare the kinetics obtained using the two methods. We were, however, able to observe that the decrease in the fluorescence of the foci detected by SOID proceeded in a slower manner with respect to the decrease in the number of foci, as demonstrated by the higher SOID values 2 and 6 hours after irradiation, mainly due to the increase over time of the size of the foci (Figure S1). We also measured the kinetics of γ-H2AX fluorescence using flow cytometry, which is a convenient method to analyze a high number of cells. In our experiments the method did not, however, prove to be sensitive enough to detect differences in γ-H2AX FI at the different time-points after irradiation with the lower IR dose (Figure S2). On the basis of our experiments, we concluded that the SOID parameter was the most useful method to evaluate both high and low values of γ-H2AX FI, reflecting the amount and the persistence of DNA damage signal.

### DSB Resolution Throughout Cell Cycle

**Figure 2:**
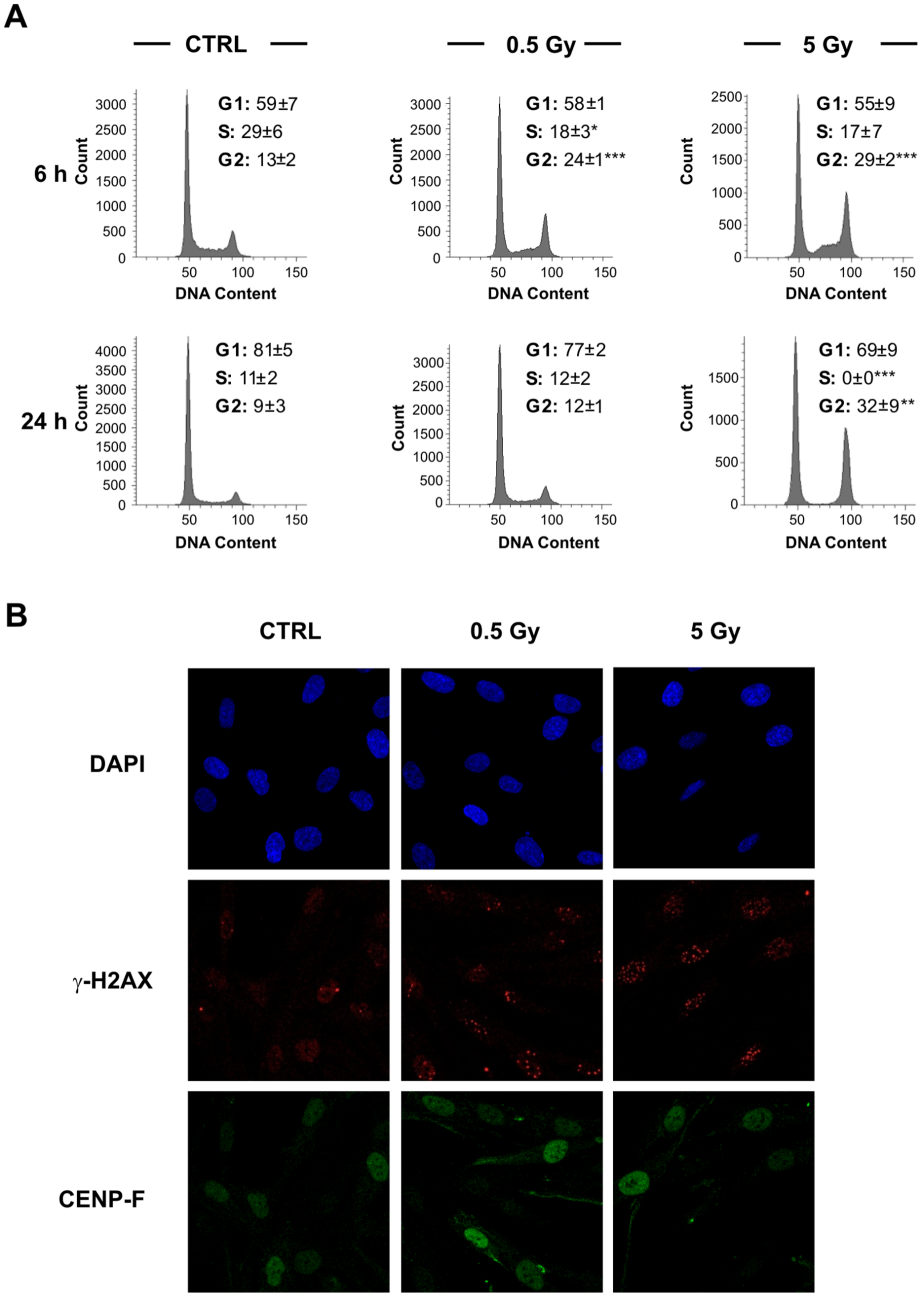
Cell cycle distribution of CCD-34Lu cells at 6 and 24h from irradiation with 0.5 and 5 Gy of γ-rays. (A) X axis, DNA content; Y axis, number of cells.*P<0.05; **P<0.01; ***P<0.001 (Student’s *t*-test). (B) Pictures of γ-H2AX foci (red) in nuclei counterstained with DAPI (blue) in CENP-F positive (green) and negative cells at 2h from irradiation with 0.5 and 5 Gy of γ-rays.

Before analyzing the efficiency of DSB rejoining during the different cell cycle phases, we performed flow-cytometry analyses of exponentially growing CCD-34Lu cells irradiated with 0.5 and 5 Gy of γ-rays. Six hours after irradiation with the two doses the fraction of S-phase cells decreased while that of G2-phase cells significantly increased (*P*<0.001, Figure 2A). Twenty-four hours after irradiation the cells irradiated with the lower dose showed a cell cycle distribution similar to that of the non-irradiated cells, while the cells irradiated with the higher dose were still blocked in the G2-phase (32% vs. 9% of control cells; *P*<0.01) and S-phase cells were completely absent. The irradiated cell samples with a diploid DNA content were only G2-phase cells, as microscopic inspection did not uncover any mitosis (data not shown). These results indicate that IR activated cell cycle checkpoints induce an arrest in G2-phase whose persistence is related to the dose of irradiation.

**Figure 3:**
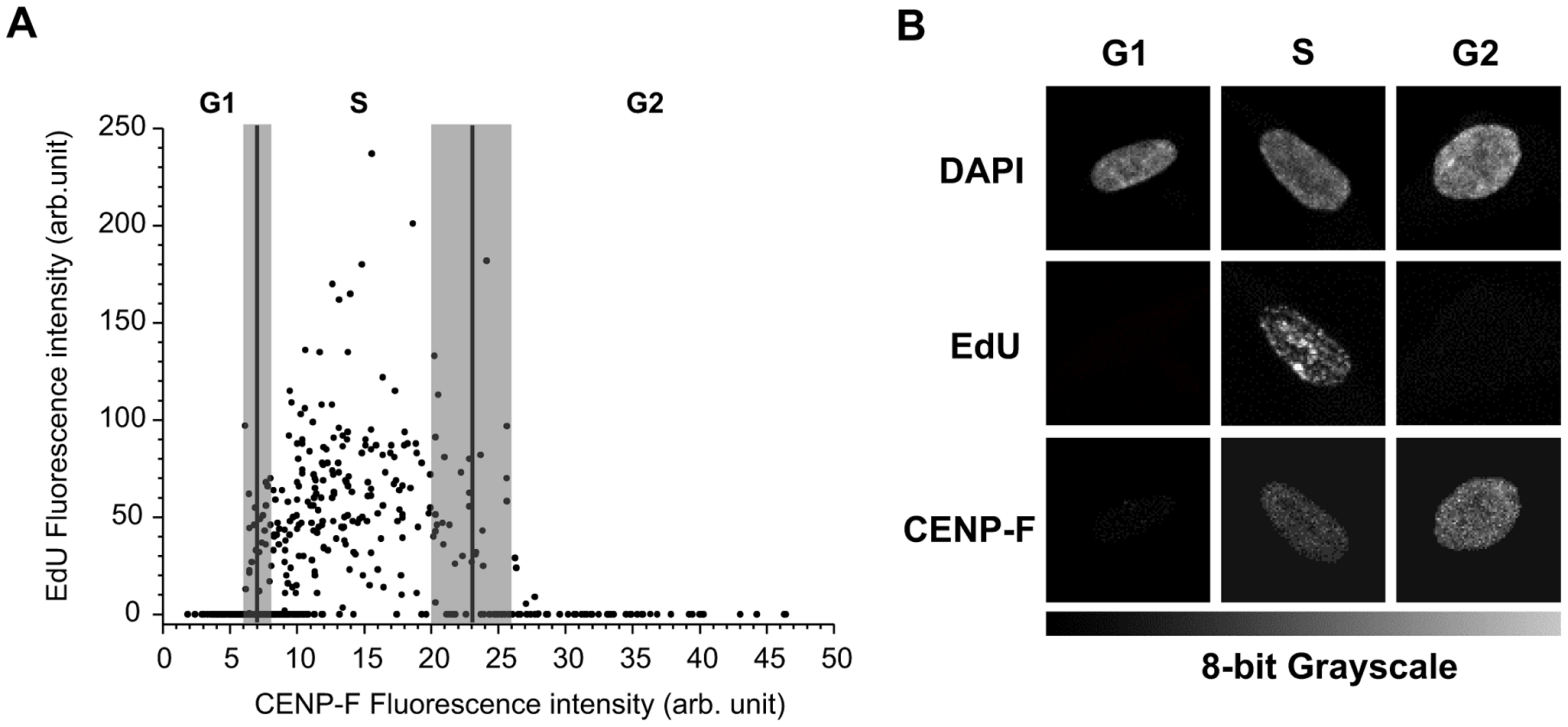
Fluorescence intensity (FI) of CENP-F protein plotted against FI of EdU. (A) CENP-F FI <6.7±0.5 has been assigned to G1 cells, CENP-F FI >22.7±2.5 to G2 cells. FI within the range 6.7–22.7 has been assigned to S phase cells. Cells with CENP-F intensity values within the confidence interval of S-phase, estimated as mean values of CENP-F ± S.D, have been excluded from the analyses (shaded area). (B) Representative pictures of EDU negative, CENP-F negative G1 cells; EDU positive, CENP-F weakly positive S-phase cells; EDU negative, CENP-F positive G2 cells.

**Figure 4:**
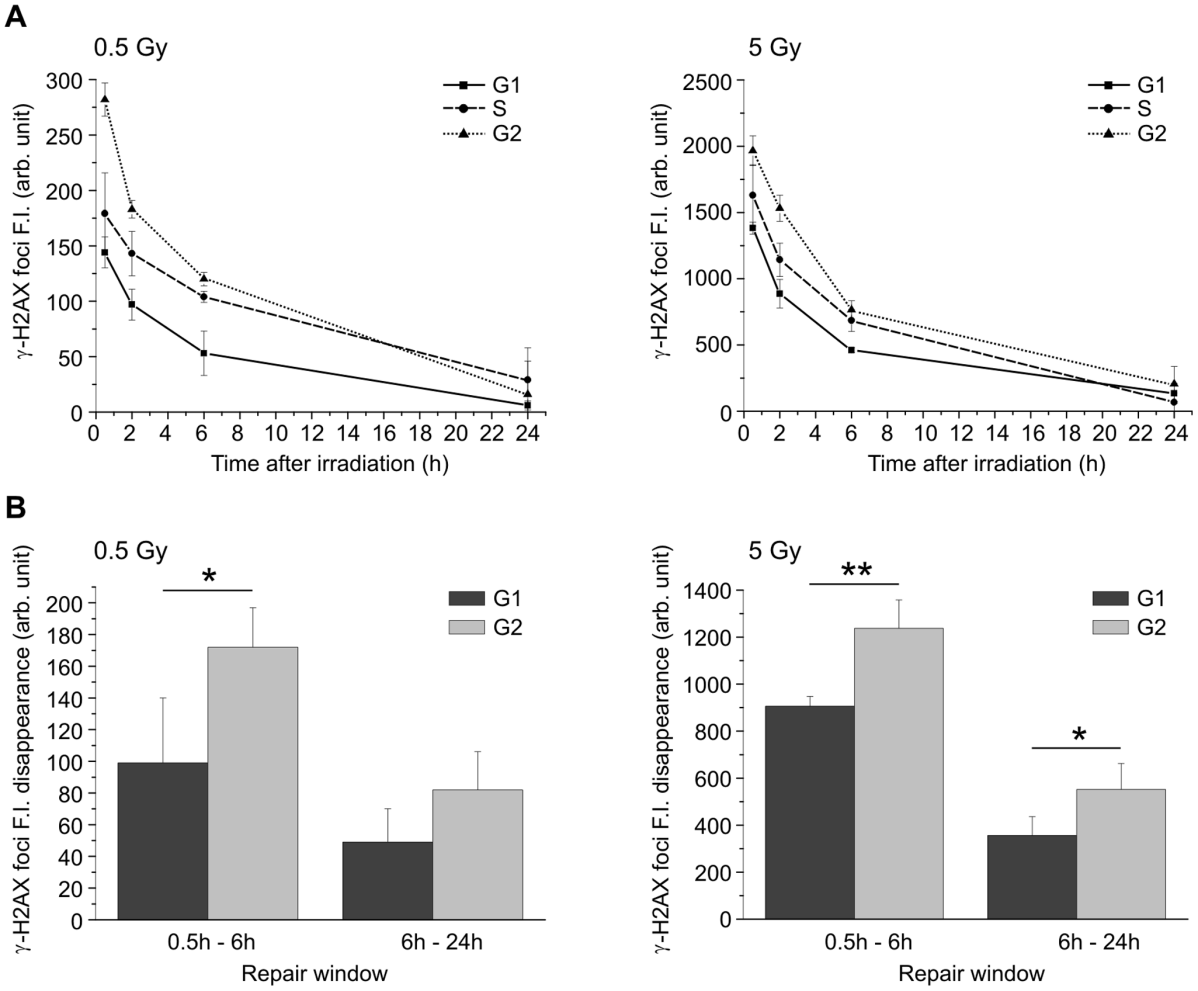
Kinetics of γ-H2AX foci in G1, S and G2 cells irradiated with γ-rays. (A) Values of γ-H2AX foci FI are mean ± S.D. from SOID determinations carried out in at least four independent experiments in cells irradiated with 0.5 and 5Gy. (B) Disappearance of γ-H2AX foci FI in G1- and G2-phases cells calculated in the 0.5–6h and 6–24h time-windows after irradiation (*P<0.05, **P<0.01, G2 cells vs. G1 cells, *t*-test).

To evaluate the efficiency of DSB rejoining in the different cell cycle phases, we used nuclear fluorescence intensity of CENP-F protein, whose expression is cell cycle-dependent, to discriminate G1, S and G2 cells. By staining the cells with CENP-F antibody, positive G2 cells can be easily distinguished from negative G1 cells, but weakly stained cells, probably in the late S-phase, cannot be reliably identified by this method (Figure 2B). To overcome this difficulty, we determined the range of CENP-F fluorescence intensity of G1, S, and G2 cells by labeling the S-phase of non-irradiated cells with EdU staining (Figure 3). Cell distribution through cell cycle phases determined with CENP-F fluorescence by confocal microscopy has been confirmed by propidium iodide FACS analysis (Figure S3). By using SOID parameter, we were thus able to monitor the kinetics of DSB resolution, based on the disappearance of damage signaling, in G1, S, and G2 cells irradiated with 0.5 and 5 Gy of γ-rays. Our results showed that the majority of IR-induced DSBs were repaired within the first 6 hours of irradiation in all cell cycle phases (Figure 4A). Moreover, at 6 hours following irradiation, the decrease in fluorescence intensity in γ-H2AX foci induced by both IR doses was higher in the G2-phase with respect to the G1-phase cells. In the subsequent post-irradiation period (6–24h), FI disappearance was similar in the G1 and G2 cells after irradiation with 0.5 Gy, but it was higher in G2 cells after irradiation with 5 Gy (Figure 4B). As shown in Figure S1A, the foci size increased similarly over time in both G1 and G2 cells, while the intensity of foci fluorescence increased over time only in the G2 cells, probably due to the persistence of some unrepaired DSBs that were formed in previous cell cycle phases (Figure S1B). These data were confirmed by FACS analyses carried out in cells irradiated with 5 Gy in which FI disappearance in the 0.5–6h time-window was higher in the G2- with respect to the G1-phase cells (*P*<0.001, G2 vs. G1; Figure S4).

**Figure 5:**
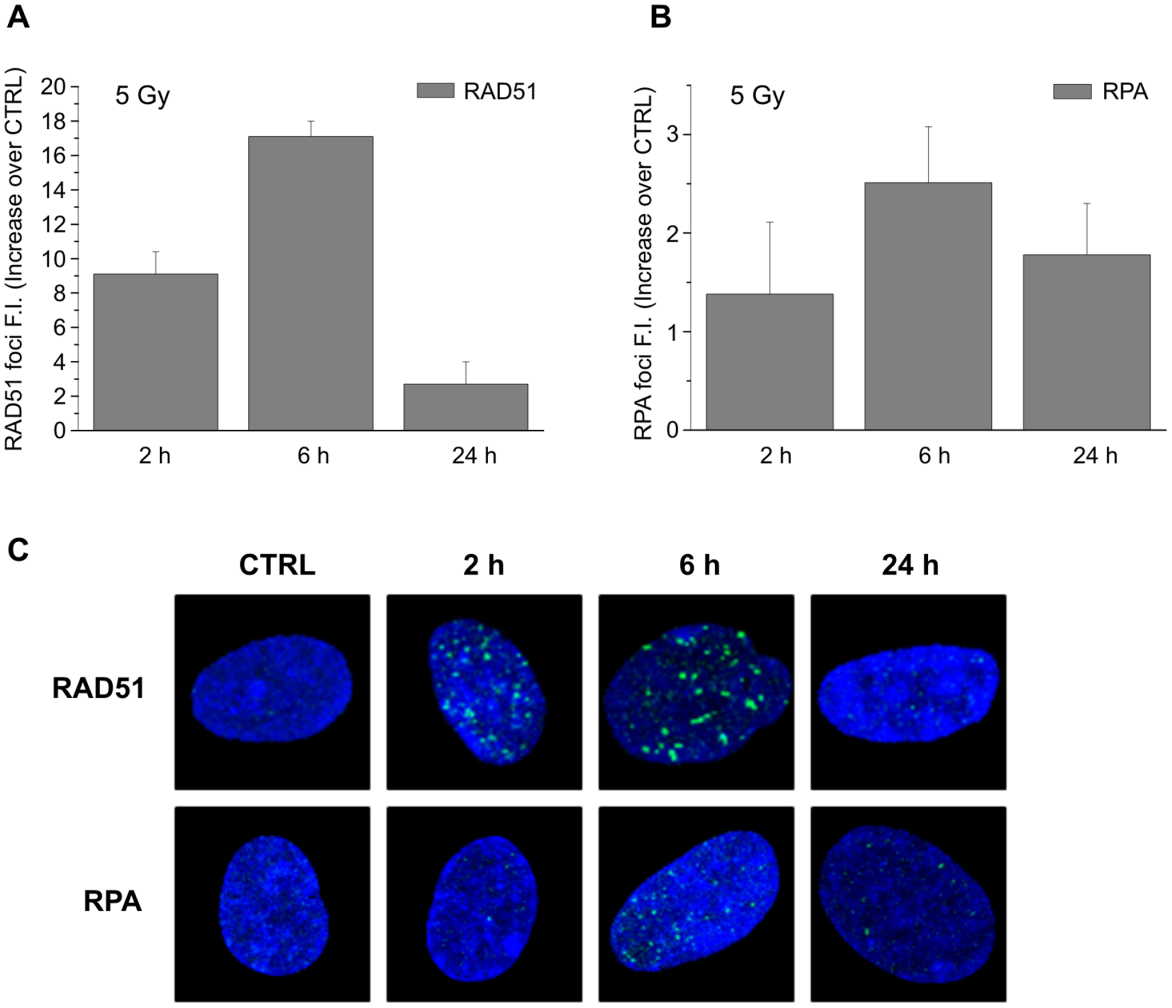
Analysis of RAD51 and RPA recruitment in CCD-34Lu cells irradiated with γ-rays. Fluorescence intensity (FI) of foci was determined at 2, 6 and 24h after irradiation with 5Gy. Values of FI are mean ± S.D. from SOID determinations carried out in at least three independent experiments and plotted as increase over controls.

Since our results suggest that DSBs are repaired faster in the G2-phase cells during the first time-window after irradiation (0.5–6h), we analyzed the kinetics of RAD51 and RPA foci formation, representative of HR repair system involvement, in CCD-34Lu cells irradiated with 5 Gy (Figure 5). RAD51 foci were clearly visible beginning 2 hours after irradiation, they peaked at 6h, and at 24h they were mostly disassembled. Foci of RPA displayed similar kinetics, with fluorescence intensity values lower than those of RAD51. We can thus conclude that some HR proteins are recruited at DSB sites during the first hours after irradiation.

**Figure 6:**
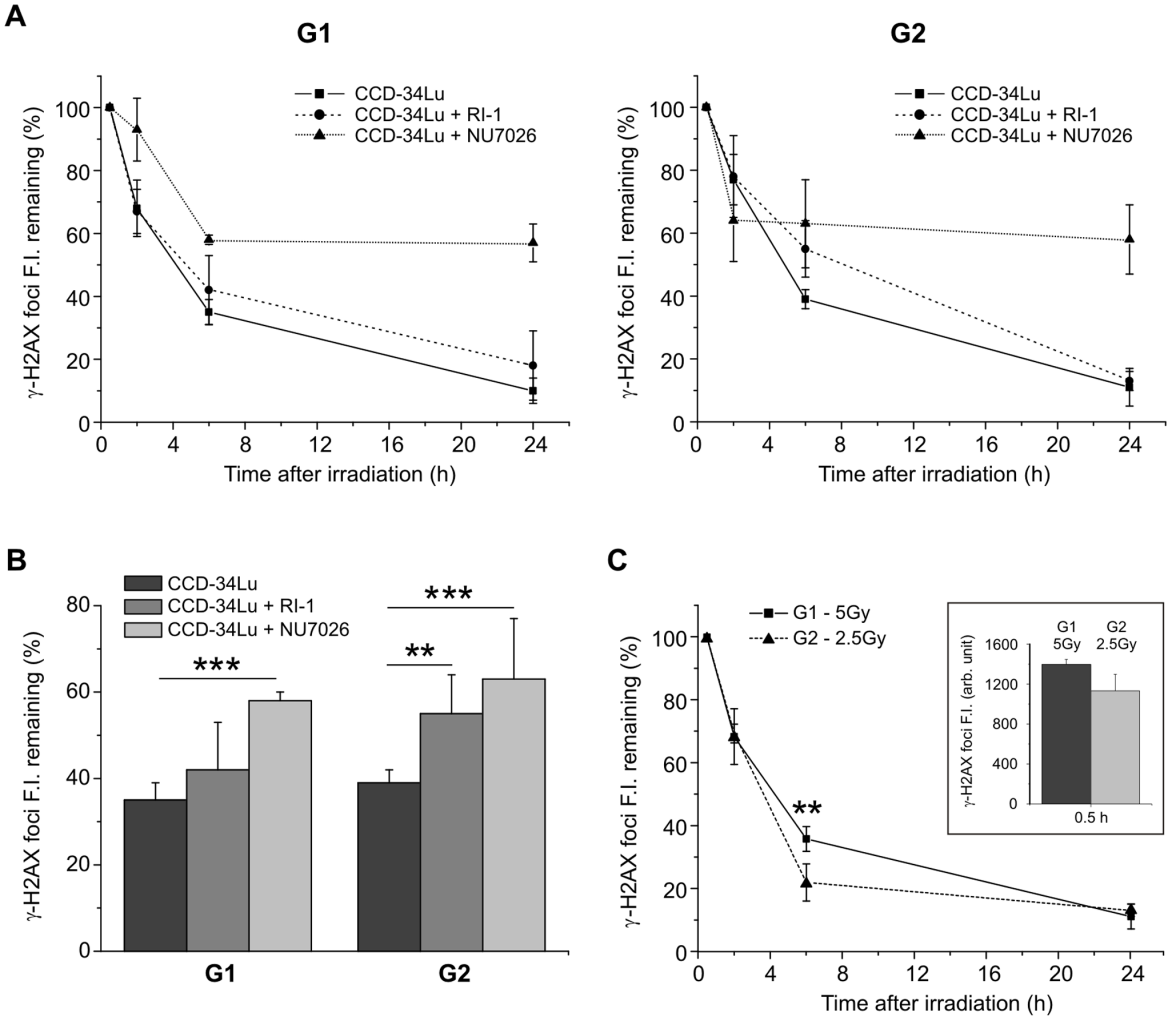
Contribution of HR and NHEJ pathways to DSB repair in G1- and G2-phases. (A) The quantification of γ-H2AX FI induced by 5 Gy was performed by SOID parameter in untreated CCD-34Lu and in NHEJ- and HR-inhibited cells, incubated respectively with the inhibitor of DNA-PKcs, NU7026, and the inhibitor of RAD51, RI-1. (B) In G1 cells the remaining FI at 6h after irradiation with 5 Gy increased in NHEJ-inhibited cells with respect to untreated cells (58% vs. 35%, ***P<0.001, *t*-test). In G2 cells both inhibitors significantly increased the remaining FI at 6h after irradiation with respect to untreated cells (in NU7026-treèated cells FI was 63% vs. 39% in untreated cells and 55% in RI-1 treated cells, **P<0.01, *t*-test). (C) Disappearance of γ-H2AX foci FI in G1- and G2-phase of cells with similar initial SOID values obtained by irradiating the cells respectively with 5 and 2.5 Gy. The values of FI at 0.5h after irradiation were 1132±166 in G2 cells irradiated with 2.5 Gy and 1450±45 in G1 cells irradiated with 5Gy (see the box on the right). At 6h after irradiation the remaining γ-H2AX foci FI was significantly higher in G2 cells (**P<0.01, *t*-test).

We then evaluated the contribution of HR and NHEJ in rejoining IR-induced DSBs in G1- and G2-phase cells by quantifying γ-H2AX foci FI in CCD-34Lu treated with NHEJ and HR inhibitors, NU7026 and RI-1, before irradiation with 5Gy. As previously shown, the majority of DSBs (∼60%) in untreated CCD-34Lu cells were repaired during the first 6 hours after irradiation both in the G1- and G2- phases (Figure 4), while HR- and NHEJ-inhibited cells showed a delay in DSB resolution in both phases (Figure 6A). In particular, six hours after irradiation the remaining FI in G1-phase cells was significantly higher in the NU7026-treated cells compared to the untreated ones (*P*<0.001, Figure 6B). After treatment with RI-1 inhibitor, instead, the percentage of remaining γ-H2AX FI was similar to that in the untreated cells. Both inhibitors significantly affected the repair of DSBs in G2-phase cells by increasing the fraction of remaining γ-H2AX FI (*P*<0.01, Figure 6B). Since DSB repair kinetics have been analyzed in cells that probably have different amounts of initial DNA damage, we analyzed DSB rejoining of γ-H2AX foci in G1 cells irradiated with 5 Gy and in G2 cells irradiated with 2.5 Gy. The initial values of SOID parameter for γ-H2AX foci in G1 and G2-phase cells were very similar in these experimental conditions: 1450±45 for G1 cells and 1132±166 for G2 cells. The results outlined in Figure 6C confirmed that the decrease of γ-H2AX foci FI was slightly but significantly higher in the G2 cells compared to the G1 cells (*P*<0.01).

### DSB Rejoining in DNA-PKcs-deficient Cells

**Figure 7:**
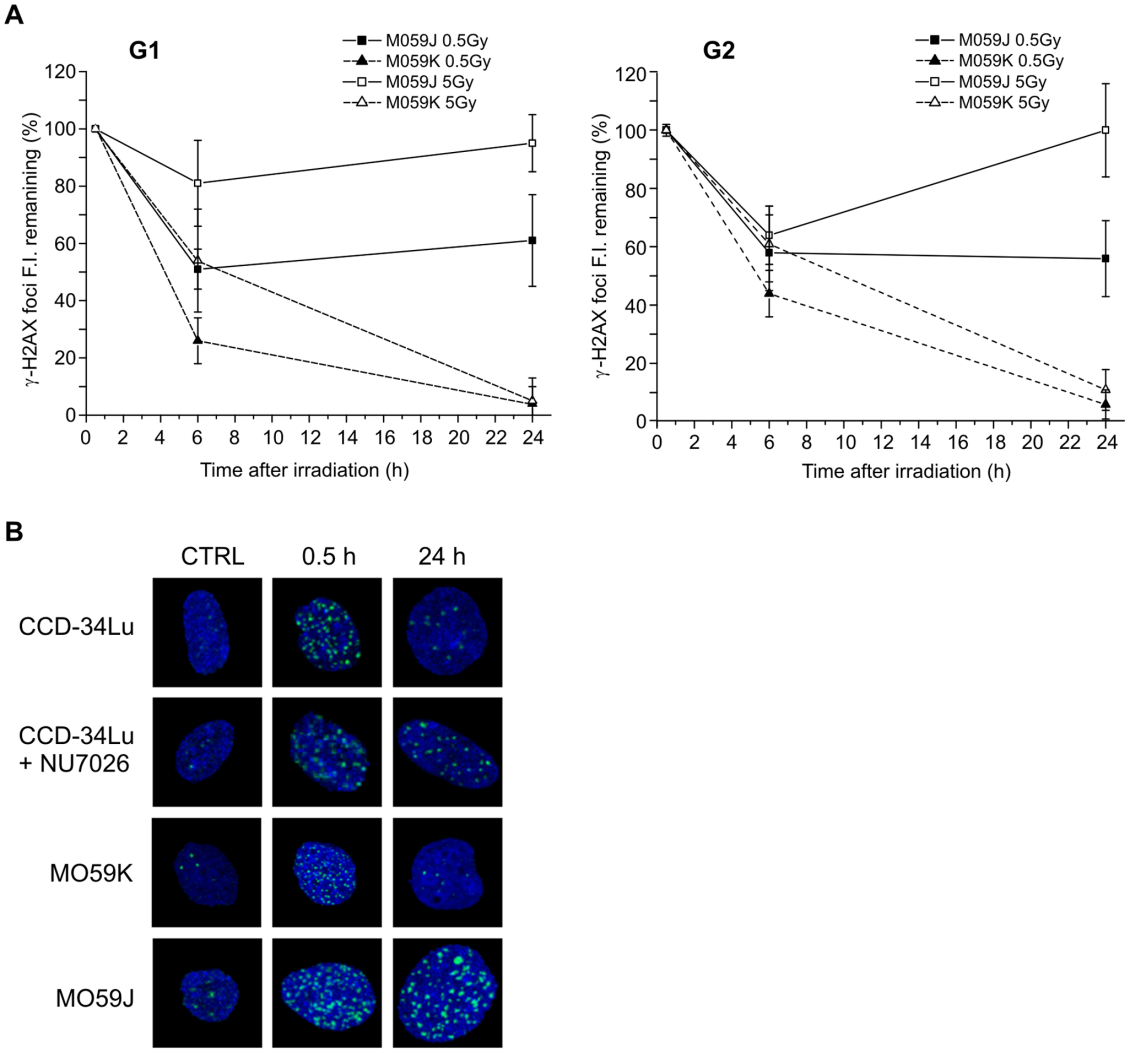
DSB repair in cells with DNA-PKcs inhibited or absent. (A) Fluorescence intensity (FI) of the remaining γ-H2AX foci was measured in G1- and G2-phase of M059K and M059J cells irradiated with 0.5 and 5 Gy of γ-rays. (B) Representative pictures of γ-H2AX foci in CCD-34Lu cells, with and without inhibitor, and in M059K and M059J cells at 0.5h and 24h after irradiation with 0.5Gy.

After we verified that the chemical inhibition of DNA-PKcs in CCD-34Lu cells affected the rejoining of DSBs in the G1 and G2 cell cycle phases, we analyzed the DNA repair kinetics in the DNA-PKcs-deficient M059J cells, and, for comparison, in the isogenic DNA-PKcs proficient M059K cell line. As reported in the literature [31] and detected in our experiments (not shown), both cell lines are G1/S checkpoint deficient. By contrast, and differently from M059K cells, M059J retained the G2/M checkpoint, as shown by the significant increase in G2-phase cells 24 hours after irradiation with 5 Gy (Figure S5). Figure 7 shows that M059K cells rejoined almost completely the DSBs induced by 0.5 and 5 Gy during the 24h of post-irradiation incubation, as was similarly observed in CCD-34Lu (Figure 6A). In M059J cells, both in G1- and G2-phases, almost 60% of γ-H2AX foci fluorescence persisted 24 hours after irradiation with 0.5 Gy, but there was no fluorescence decrease after irradiation with 5Gy.

**Figure 8:**
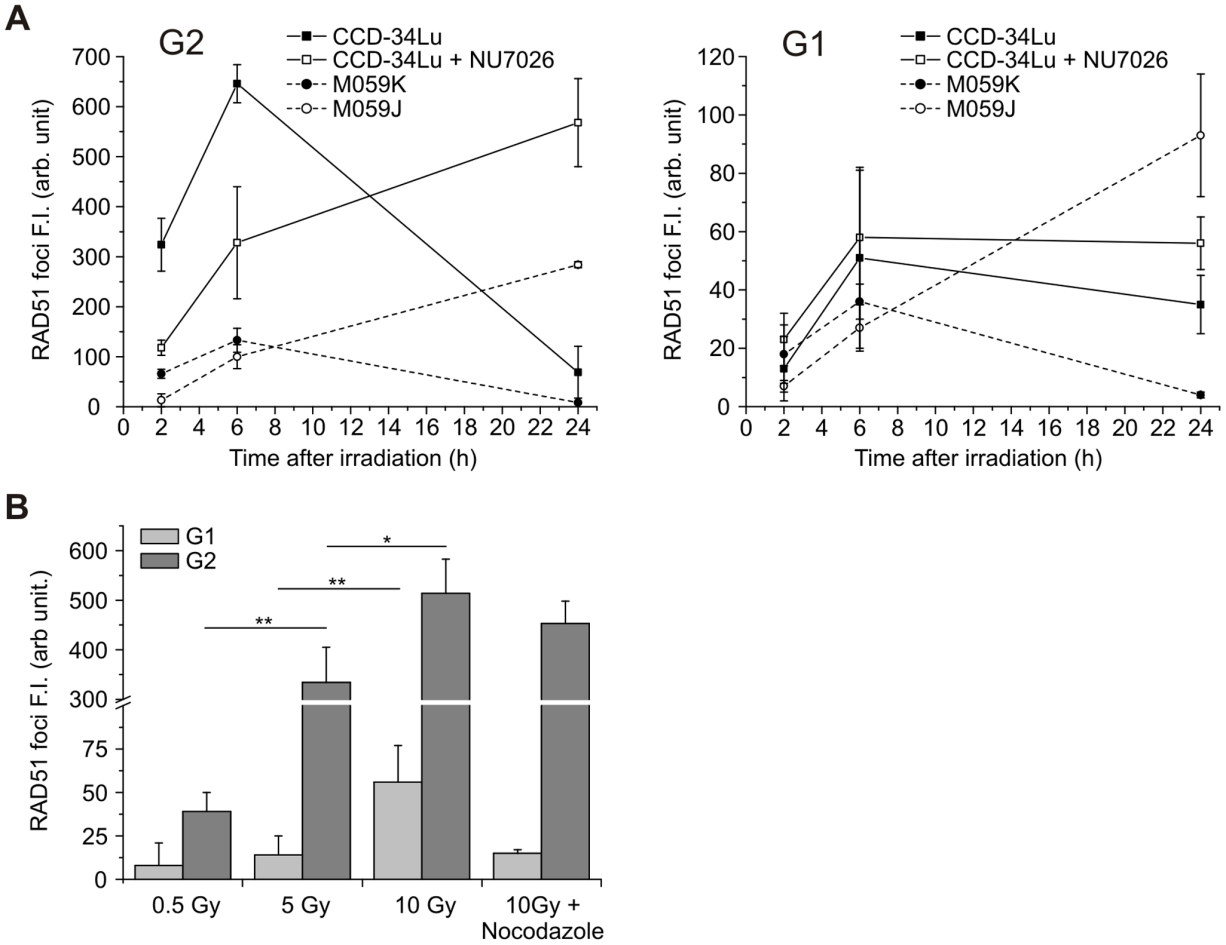
Kinetics of RAD51 foci in G1- and G2-phase. (A) RAD51 foci fluorescence intensity (FI) in CCD-34Lu, CCD-34Lu+NU7026, M059K and M059J cells irradiated with 5 Gy of γ-rays cells. (B) RAD51 foci FI in CCD-34Lu cells at 2h after irradiation with 0.5, 5 and 10 Gy; in G2 cells RAD51 recruitment significantly increases with dose (*P<0.05, **P<0.01), in G1 cells at 10 Gy RAD51 recruitment significantly increases respect to 5 Gy (**P<0.01, *t*-test). In cells treated with nocodazole before irradiation with 10 Gy no G1 cells with RAD51 foci were detected.

We analyzed the kinetics of RAD51 foci in CCD-34Lu cells incubated with the DNA-PKcs inhibitor and in M059J cells to verify if the impairment of NHEJ can alter the recruitment of HR proteins at DSB sites (Figure 8). In cells proficient for DNA-PKcs (i.e. untreated CCD-34Lu cells and M059K cells) the fluorescence intensity of RAD51 foci after irradiation with 5Gy peaked at 6h and then fell at 24h in both the G1 and G2 cells. On the contrary, in NU7026-treated CCD-34Lu and in M059J cells the formation of RAD51 foci in the G2-phase was strongly affected by the absence of DNA-PKcs activity, with an initial delay in the RAD51 recruitment, followed by a progressive increase in foci FI up to 24h. The kinetics of RAD51 foci was similar in NU7026-treated and untreated CCD-34Lu cells in G1-phase, while it was very different in M059J and M059K cells, which was true also in the G2-phase (Figure 8A).

We also evaluated if the radiation dose alters regulation of the choice between NHEJ and HR repair systems during cell cycle progression. FI of RAD51 foci was quantified two hours after CCD-34Lu cells were irradiated with increasing doses of γ-rays. We chose to quantify the foci at that time point because, as described by other investigators and indicated by our previous observations (our unpublished results and [22], [32], [33]), a peak in RAD51 foci induction was noted between one to 4 hours following irradiation.

As expected, the recruitment of RAD51 at the sites of DSBs significantly increased with the γ-ray dose in the G2 cells as well as after irradiation with 10 Gy, also in G1 cells (Figure 8B). The quantification of RPA foci FI was in agreement with that of RAD51 in G2 cells, while there was a negative or very scanty recruitment of RPA in G1 cells irradiated with 5–10 Gy (data not shown). To check the origin of RAD51 loaded at the DBS sites of G1 cells irradiated with 10 Gy, we blocked cell-cycle progression from G2/M to G1-phase using nocodazole, the inhibitor of the mitotic spindle. Under these experimental conditions, no G1 cells with RAD51 foci were detected, indicating that the foci previously observed were probably due to the persistence of unrepaired DSBs formed in the G2-phase of cells that 2h later passed to G1-phase.

**Figure 9:**
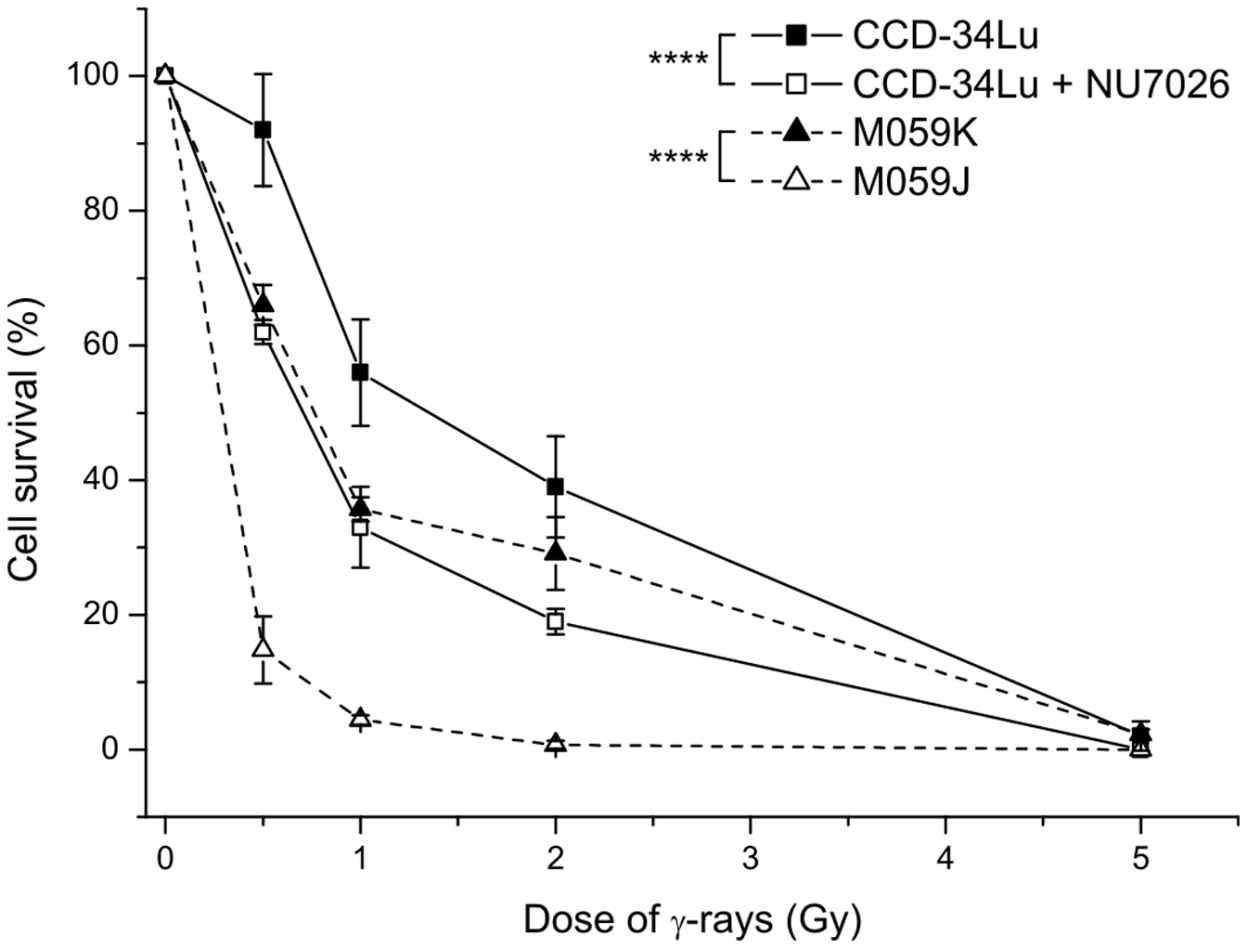
Cell survival after irradiation with increasing doses of γ-rays. Cells impaired for NHEJ repair pathway showed a significant lower survival at 0.5, 1 and 2 Gy respect to their proper control cells (CCD-34Lu+NU7026 vs. CCD-34Lu cells and M059J vs. M059K cells, ****P<0.0001, two-way ANOVA). Data are means ± S.D. of 3–5 independent experiments.

The decrease in DNA repair efficiency in DNA-PKcs-inhibited CCD-43Lu and in DNA-PKcs-deficient M059J cells markedly affected cell viability after irradiation with increasing IR doses (Figure 9). Cell survival of NU7026-treated CCD-34Lu was significantly lower than that of the untreated cells for all the doses (*P*<0.0001). Similar results were observed in the M059J with respect to the DNA-PK proficient M059K cells (*P*<0.0001). At the lowest dose of γ-rays (0.5 Gy), the viability of untreated CCD-34Lu was slightly affected, while it was significantly lower than that in non-irradiated cells when incubated with the NU7026 inhibitor (62% vs. 92% ). The same γ-ray dose reduced the survival of M059K to 65% and that of M059J to 15% with respect to the non-irradiated cells.

## Discussion

Increasing doses of γ-rays were utilized to investigate if DNA damage of different structural complexities affects DSB signaling and repair. The questions of whether and to what extent HR or NHEJ are involved in repairing radio-induced DSBs were also addressed. The shift from NHEJ toward HR as the cell cycle progresses from G1 to S/G2 is regulated by CDK activity. It has been seen that during homologous recombination repair process CDK-mediated phosphorylation at ser3291 of BRCA2 blocks its interaction with the RAD51 protein required for homology searching and strand invasion. HR is, thus, blocked when ssDNA resection is prevented by CDK1 inhibition and early during G1-phase when CDK1 is inactive. Some investigators have reported that in cells exposed to IR cell cycle checkpoint control can be bypassed, leading to a rapid decrease in ser3291 phosphorylation and an increased association of BRCA2 with RAD51 to promote DSB repair by HR [18], [34], [35].

In agreement with previous data demonstrating that the time course of 53BP1 foci formation and disappearance is similar to that of γ-H2AX foci, our results in human fibroblasts irradiated with 0.5 Gy of γ-rays indicate that the kinetics of γ-H2AX and 53BP1 foci is very similar (Figure 1) [10]–[14]. To verify whether DSB repair efficiency was dose-related, the kinetics of γ-H2AX cellular content after irradiation with increasing doses of γ-rays was assessed using different methods: manual counting of nuclear foci, fluorescence intensity of foci measured by the SOID parameter and flow cytometry. In agreement with Ishikawa et al.’s findings, our results indicated that the SOID parameter is an accurate method to quantify even slight variations of γ-H2AX fluorescence [26]. By contrast, γ-H2AX kinetics after 5 Gy irradiation could not be evaluated by manual foci counting since the number of foci induced by that dose was too high for a reliable determination; nor does the method take into account the increase in foci size detected at later times after irradiation. When the kinetics of DSB rejoining was analyzed by means of the SOID parameter, it was possible to quantify not only the number of the remaining foci but also the total fluorescence of DDR proteins still associated to DSBs. In particular, the size increase of γ-H2AX foci over time markedly influences the SOID parameter value, highlighting the persistence of some DSBs difficult to repair during the post-irradiation incubation.

By comparing the efficiency in rejoining radiation-induced DSBs during cell cycle progression, we found that in G2-phase cells, whose frequency significantly increased after irradiation (Figure 2A), DNA repair proceeded very efficiently, both during the first period of repair-incubation (0.5–6h) and at later times (6-24h, Figure 4). Previous studies reported that the kinetics of IR-induced DSB repair in the G1-phase exhibit fast as well as slow components [36], [37]. The former removes the majority of DSBs within the first 2 h, while the latter represents a sub-pathway of the NHEJ pathway [38], [39]. Just as in G1, DSB-repair kinetics are biphasic in G2-phase with the fast component representing NHEJ and accounting for the majority of DSB-repair events, while the slow component represents HR and accounts for 15–20% of DSB-repair events [22]. We analyzed the kinetics of γ-H2AX foci in the G1- and G2-phases of HR-inhibited cells to evaluate the contribution of HR in rejoining DNA DSBs during cell cycle progression. According to our results, G2-phase cells treated with the HR inhibitor (RI-1) exhibited a higher level of unrepaired DSBs 6 hours after irradiation (Figure 6). By comparing the repair rate in G1 and G2 phase cells having a similar amount of initial DNA damage, we were able to confirm that the HR repair system contributed to rejoining DSBs also during the first hours following irradiation (Figure 6C), differently from what reported by Beucher et al. [22]. The differences between our and Beucher’s findings could be explained by the two different methods adopted to measure the decreased level of γ-H2AX: counting the number of foci used by Beucher [22] and the SOID parameter used during our experiments. The hypothesis that HR is involved in the first hours after IR is, moreover, supported by recent observations by Gandhi et al., [40] who demonstrated that within 5 min of irradiation, homologous chromosomes make contact at the sites of DSBs induced by ionizing radiation in human G0–G1 cells.

Proteins that mediate the recruitment of HR and NHEJ are generally distinct, but in some cases they are implicated in both pathways. Among those proteins, DNA-PK primarily regulates DSB repair by NHEJ, but it can also influence HR. Several studies have reported that when DNA-PKcs is absent and NHEJ is compromised, HR repair is enhanced [41]. Other authors have, instead, reported that HR involvement is reduced during DNA repair of cells in which DNA-PKcs is physically present but functionally compromised [42]. It would seem then that functionally compromised DNA-PKcs has more severe consequences than the enzyme’s complete absence, probably blocking its auto-phosphorylation that allows the NHEJ pathway to proceed [37]. Experiments on CCD34-Lu cell line in which DNA-PKcs was impaired through chemical inhibition (NU7026) and DNA-PK deficient M059J cells as a consequence of a nonsense frame-shift mutation [43] were carried out to evaluate contribution of HR in DSB repair. The presence of DNA-PKcs inhibitor significantly decreased the efficiency of DSB rejoining in irradiated CCD-34Lu cells, both in G1- and G2-phase, as well as in M059J cells (Figures 6 and 7). Moreover, when NHEJ was compromised, the recruitment of RAD51 at DSB sites in both NU7026-treated CCD-34Lu and M059J cells was low early after irradiation and continued up to 24h in both G1 and G2 cells (Figure 8). In M059J cells, DSBs present in the G2-phase were partially caused by lesions induced in this phase of the cell cycle as well as by unrepaired lesions that occurred in the G1-phase and accumulated 24h later in the G2-phase due to the lack of a G1/S checkpoint. However, despite the extensive recruitment of RAD51 at DSB sites 24h after irradiation, the HR pathway, which should be fully functional in those cells, is unable to compensate for the impairment of NHEJ, as evidenced by the persistence of γ-H2AX phosphorylation and the low level of cell survival, even after irradiation with low doses of ionizing radiation.

As we analyzed if radiation dosage alters the choice between the NHEJ and the HR repair systems during cell cycle progression, we observed that RAD51 was recruited at DSB sites even in G1 cells irradiated with the highest dose. Although in accordance with data outlined by Kim et al. [33] and by Rapp and Greulich [44] showing the presence of RAD51 foci in the G1-phase of irradiated cells, these observations can be more accurately interpreted by blocking the transition of cells from the G2/M- to the G1-phase. Indeed, cells irradiated with high doses of IR in the G2-phase could be able to progress to the G1-phase with unrepaired DSBs. By blocking the progression of G2 cells with unrepaired DSBs, we demonstrated that the presence of G1 cells positive for RAD51 foci can be explained by the advancement of cells with RAD51 foci formed in the S-G2 phases.

Our data generally agree with the model that NHEJ is the major pathway for IR-induced DSBs repair. They also demonstrate that RAD51, the main protein in the HR pathway, participates, together with NHEJ, in DSB rejoining even during the first hours after irradiation. RAD51 recruitment and activity at DSB sites appears, however, to be strictly dependent on the integrity of NHEJ components, highlighting the dominant role that DNA-PKcs play in regulating the cell response to DNA damage throughout cell cycle progression.

## Supporting Information

Figure S1 Analysis of γ-H2AX foci physical parameters in G1 and G2 cells after irradiation with 5 Gy of γ-rays. (A) γ-H2AX foci size increased significantly with time after irradiation in both G1 and G2 phases (*P<0.05, **P<0.01, t-test) without differences between the two phases. (B) Signal intensities of γ-H2AX foci were determined by SOID parameter in the same cells. The intensity of foci fluorescence increased with time only in G2 cells (***P<0.001, *t*-test). The values are mean fluorescence intensity ± S.D. of single γ-H2AX foci determined from at least 30–50 nuclei for each time-point.
(TIF)

Figure S2 Kinetics of γ-H2AX total fluorescence determined by FACS analysis in cells irradiated with 0.5 and 5 Gy of γ-rays.
(TIF)

Figure S3 Comparison of data of cell distribution in cell cycle phases obtained from FACS analysis and from confocal microscopy of CENP-F F.I. in the same cell population.
(TIF)

Figure S4 γ-H2AX total fluorescence determined by FACS analysis. Kinetics of γ-H2AX in G1, S and G2 cells irradiated with 5 Gy of γ-rays. (B) In the time-interval 0.5–6h, the γ-H2AX FI disappearance was significantly higher in G2 vs. G1 cells (*P*<0.001, G2 vs. G1).
(TIF)

Figure S5 Cell cycle distribution in M059J and M059K cells after irradiation with 5Gy of γ-rays. In both cell lines γ-irradiation did not induce the G1/S checkpoint, whereas only in M059J cells the G2/M checkpoint was activated. Data are mean ± S.D. from three independent experiments.
(TIF)
