# Supplementary figures and images for: The Efficiency of Homologous Recombination and Non-Homologous End Joining Systems in Repairing Double-Strand Breaks during Cell Cycle Progression
Source: PLoS One. 2013 Jul 11;8(7):e69061. doi: 10.1371/journal.pone.0069061 (PMC3708908; doi:10.1371/journal.pone.0069061)

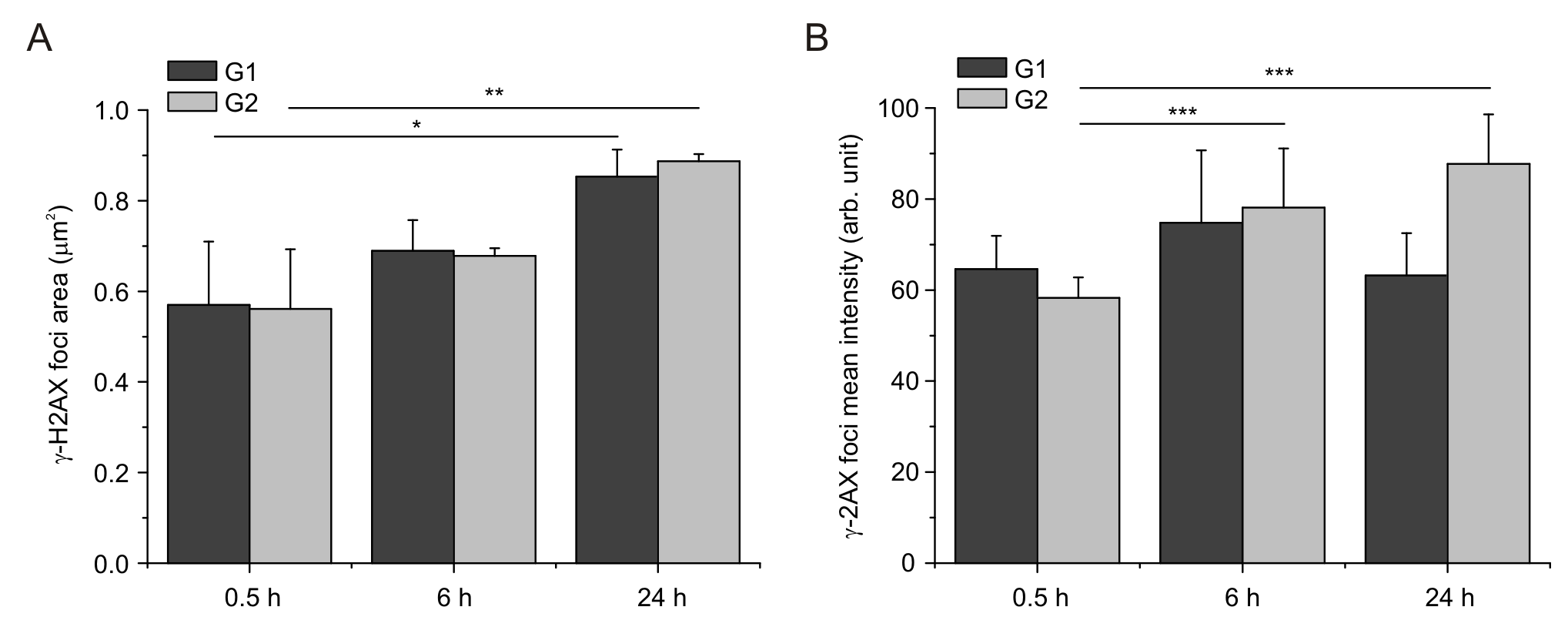

Supplement: Figure S1 — Analysis of γ-H2AX foci physical parameters in G1 and G2 cells after irradiation with 5 Gy of γ-rays. (A) γ-H2AX foci size increased significantly with time after irradiation in both G1 and G2 phases (*P<0.05, **P<0.01, t-test) without differences between the two phases. (B) Signal intensities of γ-H2AX foci were determined by SOID parameter in the same cells. The intensity of foci fluorescence increased with time only in G2 cells (***P<0.001, t-test). The values are mean fluorescence intensity ± S.D. of single γ-H2AX foci determined from at least 30–50 nuclei for each time-point. (TIF) [file pone.0069061.s001.tif]

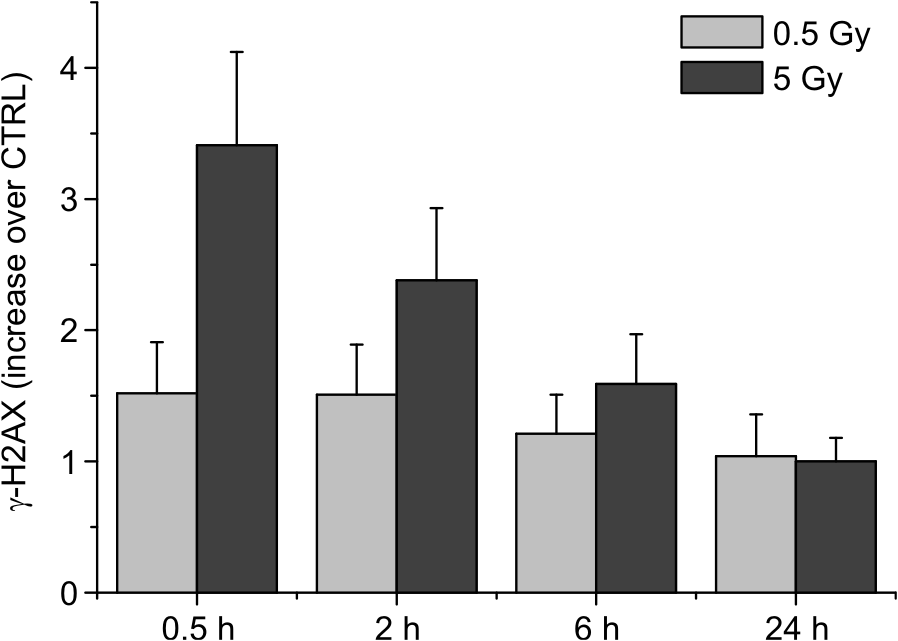

Supplement: Figure S2 — Kinetics of γ-H2AX total fluorescence determined by FACS analysis in cells irradiated with 0.5 and 5 Gy of γ-rays. (TIF) [file pone.0069061.s002.tif]

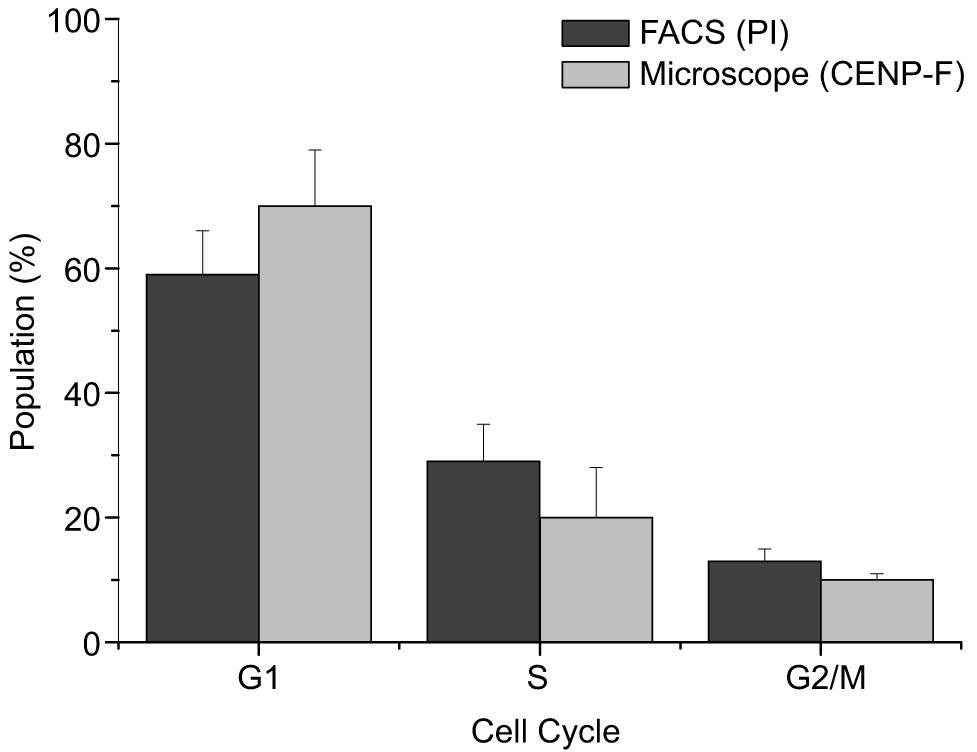

Supplement: Figure S3 — Comparison of data of cell distribution in cell cycle phases obtained from FACS analysis and from confocal microscopy of CENP-F F.I. in the same cell population. (TIF) [file pone.0069061.s003.tif]

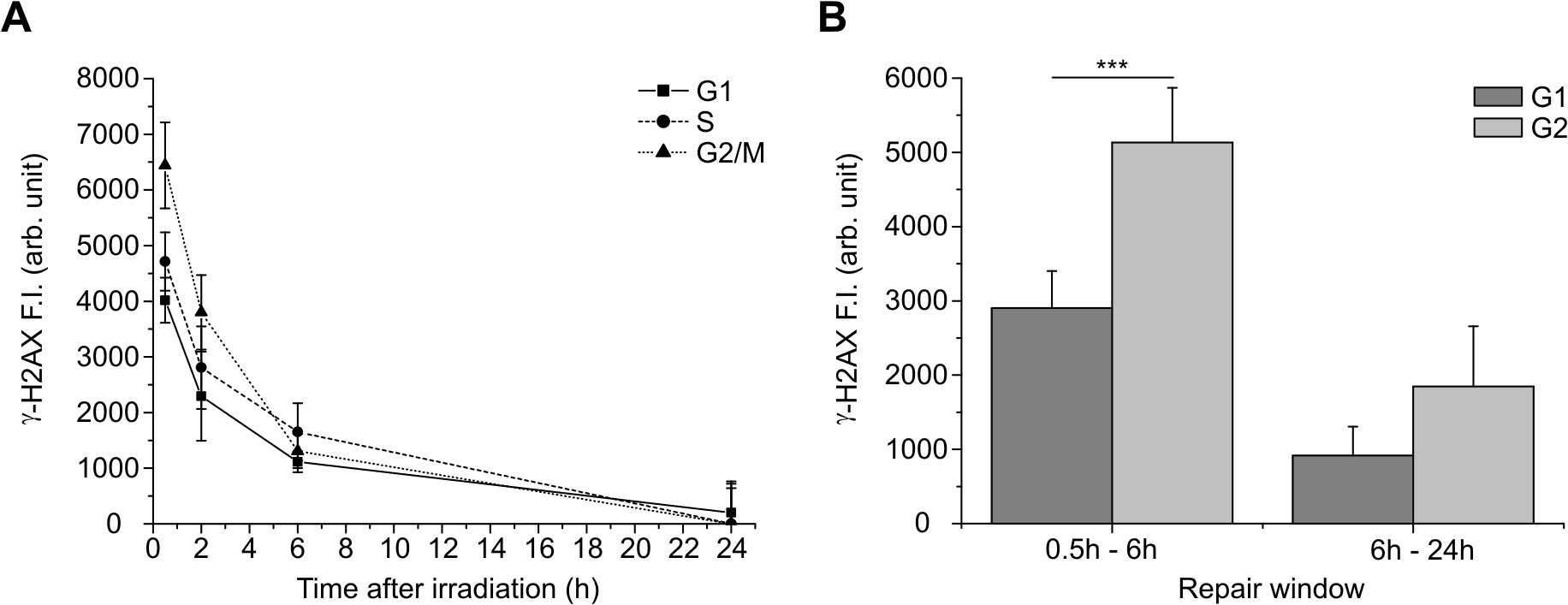

Supplement: Figure S4 — γ-H2AX total fluorescence determined by FACS analysis. Kinetics of γ-H2AX in G1, S and G2 cells irradiated with 5 Gy of γ-rays. (B) In the time-interval 0.5–6h, the γ-H2AX FI disappearance was significantly higher in G2 vs. G1 cells (P<0.001, G2 vs. G1). (TIF) [file pone.0069061.s004.tif]

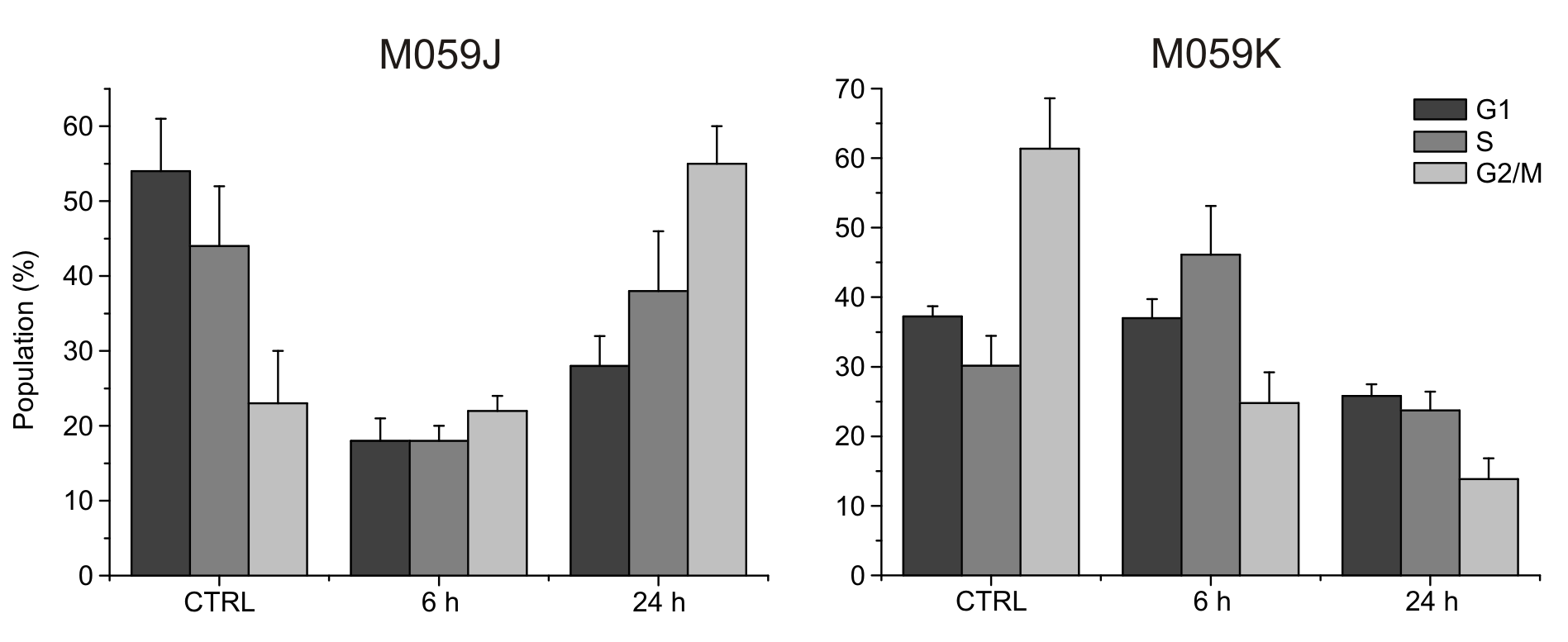

Supplement: Figure S5 — Cell cycle distribution in M059J and M059K cells after irradiation with 5Gy of γ-rays. In both cell lines γ-irradiation did not induce the G1/S checkpoint, whereas only in M059J cells the G2/M checkpoint was activated. Data are mean ± S.D. from three independent experiments. (TIF) [file pone.0069061.s005.tif]
